# Preliminary Transcriptome Analysis of Long Noncoding RNA in Hypothalamic-Pituitary-Mammary Gland Axis of Dairy Cows under Heat Stress

**DOI:** 10.3390/biom13020390

**Published:** 2023-02-18

**Authors:** Hanfang Zeng, Shujie Li, Yunfei Zhai, Haomiao Chang, Zhaoyu Han

**Affiliations:** College of Animal Science and Technology, Nanjing Agricultural University, Nanjing 210095, China

**Keywords:** dairy cow, heat stress, hypothalamic-pituitary-mammary gland axis, lncRNA, ceRNA network

## Abstract

Heat stress (HS) is directly correlated to mammary gland dysfunction in dairy cows, especially in summer. The hypothalamic−pituitary−mammary gland axis (HPM axis) plays an important role in the regulation of stress response and lactation physiology in heat−stressed dairy cows. The aim of this study was to explore the lncRNA profile, and the competitive endogenous RNA (ceRNA) regulatory network in hypothalamus, pituitary, and mammary gland tissues of heat−stressed and normal dairy cows. We performed RNA sequencing (RNA−seq) to identify differentially expressed (DE) lncRNAs, and the ceRNA regulatory network was established in HPM−axis−related tissues. Our results showed that a total of 13, 702 and 202 DE lncRNAs were identified in hypothalamus, pituitary, and mammary glands, respectively. Of lncRNAs, 8, 209 and 45 were up−regulated, and 5, 493 and 157 lncRNAs were down−regulated. Gene ontology (GO) and Kyoto Encyclopedia of Genes and Genomes (KEGG) enrichment analyses indicated that DE lncRNAs target genes that might play a role in hormone synthesis, secretion and action, apoptosis, mitogen−activated protein kinase (MAPK), AMP−activated protein kinase (AMPK), and mechanistic target of rapamycin (mTOR) signaling pathway. Moreover, the ceRNA regulatory network associated with the MAPK signaling pathway in HPM−axis−related tissues contains 3286 lncRNA–mRNA pairs. Furthermore, the ceRNA regulatory network associated with apoptosis, prolactin, AMPK, and mTOR signaling pathway in the mammary gland contains 772 lncRNA–mRNA pairs. Thus, some lncRNAs may be involved in the regulation of stress response and the physiological process of lactation. The changes in lncRNA expression profiles and ceRNAs (lncRNA–miRNA–mRNA) in HPM−axis−related tissues are the key to affect the stress response and lactation physiology of dairy cows under HS, which provide a theoretical basis for the molecular mechanism in the stress response of HPM−axis−related tissues in dairy cows under HS.

## 1. Introduction

A series of stress reactions of dairy cows caused by high−temperature environments have been widely studied. The cumulative research shows that heat stress causes the reduction of feed intake, lactation performance, and reproductive performance of dairy cows, as well as immune dysfunction, which directly and/or indirectly affects the health of dairy cows [1]. As a major production index of dairy cows, lactation performance is easily affected by heat stress in summer [2]. Presently, cows cope with heat stress in summer mainly through physical cooling and dietary nutrition changes [3,4]. However, the hypothalamic-pituitary-endocrine axis regulates the homeostasis imbalance caused by environmental thermal stimulation [5]. Among them, hypothalamic-pituitary-mammary gland axis (HPM axis) plays an essential role in stress response and physiological regulation of lactation [6]. For example, prolactin (PRL) and growth hormone (GH) secreted by the pituitary gland are closely related to the development and lactation of mammary glands. Accordingly, it is particularly important to analyze the molecular mechanism of heat stress regulating the HPM axis in dairy cows for improving their lactation performance.

Long noncoding RNA (lncRNA) is a type of RNA that does not have the ability to encode proteins and is no more than 200 nucleotides in length. As an important part of gene transcription, lncRNA can affect the expression of functional genes through transcriptional regulation and posttranscriptional regulation [7]. Recently, lncRNA has been considered as having an essential role in mammary gland development and lactation [8]. Zheng et al. profiled the transcriptome of the mammary gland using RNA sequencing (RNA−seq) technology at peak and late lactation stages of Holstein cows, and 12 differentially expressed (DE) lncRNA potentially played important roles in bovine lactation [9]. The lncRNA mammary proliferation and fatty acid synthesis−associated transcript (MPFAST) is highly expressed in the Holstein cow mammary gland during the middle lactation period compared to the dry period, and lncRNA MPFAST regulates the expression of the genes in the phosphoinositide 3−kinase/protein kinase B (PI3K−AKT) signaling pathway through sponging miR−103 and promotes the proliferation and synthesis of fatty acids of BMECs [10]. In the past few years, accumulating evidence has indicated lncRNAs are widely involved in regulating heat stress response in animals [11,12]. Researchers found that 24,795 novel and 3763 known lncRNAs were expressed in bovine mammary gland by using RNA−seq in heat−stressed and nonheat−stressed cows [13]. However, the comprehensive analysis of the differential expression of lncRNA in the HPM axis of dairy cows under heat stress has not been reported.

Therefore, this study used RNA−seq to analyze the differential expression profile of lncRNA in the HPM axis under heat stress and determined and clarified the role of lncRNA in regulating heat−stressed dairy cows. This result provides a theoretical basis for studying the molecular mechanism of the HPM axis.

## 2. Materials and Methods

### 2.1. Animal Ethics Statement

All experimental procedures were carried out following the Institutional Animal Care and Use Committee of Nanjing Agricultural University, China (SYXK 2011−0036).

### 2.2. Animals and Sample Collection

There was continuous monitoring of temperature and humidity index (THI) of the cowshed, rectal temperature, and respiratory rate of Holstein cows under the same management level in summer (August, heat−stress group (HS), *n* = 20) and winter (December, nonheat−stress group (NHS), *n* = 20) in Jielong Animal Husbandry Co., Ltd. (Huai’an, Jiangsu, China). Information about parity, lactation days, and milk yield of the cows in the NHS group and HS group is shown in previous articles [14]. In brief, the temperature (T, °C) and relative humidity (RH, %) of the cowshed were measured at 10:00 and 20:00 every day for a month. Electronic thermometers at the six fixed points in the cowshed were read, respectively. THI criteria were derived from the NRC (1971), and THI was calculated according to THI = (1.8 × T + 32) – (0.55 – 0.55 × RH) × (1.8 × T − 26), where T is the temperature (°C) and RH is the relative humidity (%) [15]. Rectal temperature and respiratory rate were measured by animal thermometer and stopwatch, respectively, at 10:00 and 20:00 every day of the experiment. 

Six lactating Holstein cows (NHS: *n* = 3; HS: *n* = 3) were randomly selected from the NHS and HS groups. Cows were purchased and slaughtered by exsanguination, and the hypothalamus tissues (HS: HS_H1, HS_H2, and HS_H3; NHS: NHS_H1, NHS_H2, and NHS_H3), the pituitary tissues (HS: HS_P1, HS_P2, and HS_P3; NHS: NHS_P1, NHS_P2, and NHS_P3), and the mammary gland tissues (HS: HS_M1, HS_M2, and HS_M3; NHS: NHS_M1, NHS_M2, and NHS_M3) were collected and frozen in liquid nitrogen within 20 min after death for subsequent experiments [14].

### 2.3. Detection of Heat Shock Proteins (HSPs) and Antioxidant Enzyme Activity

As a marker protein of heat stress, we detected the mRNA expression of *HSPs* in HPM−axis−related tissues, including heat shock protein 27 (*HSP27*), heat shock protein 70 (*HSP70*), and heat shock protein 90 (*HSP90*). Commercial kits were used to measure concentrations of malondialdehyde (MDA, Jiancheng Biological Project, cat: A003−1−2, Nanajing, China), superoxide dismutase (SOD, Jiancheng Biological Project, cat: A001−3−1, Nanjing, China), and glutathione peroxidase (GPx, Beyotime, cat: S0058, Shanghai, China) in HPM axis tissues according to the manufacturer’s instructions. The activity of these antioxidant enzymes was calculated based on the absorbance. The experiment was repeated three times. Statistical analysis was performed using SPSS version 20 (SPSS Inc., Chicago, IL, USA). The significance of differences was determined using Student’s *t*−test, significance was set at *p* < 0.05, and all data were expressed as the mean ± SEM. 

### 2.4. Total RNA Extraction and Sequencing

We selected three tissue samples from each group for lncRNA sequencing. Total RNA was extracted using a Trizol reagent kit (Invitrogen, Carlsbad, CA, USA) according to the manufacturer’s protocol. After total RNA was extracted, the rRNAs were depleted for the construction of a total RNA sequencing library. The digested products were size selected by agarose gel electrophoresis, PCR amplified, and sequenced using an Illumina Nova−Seq 6000 by Gene Denovo Biotechnology Co. (Guangzhou, China).

### 2.5. Long Noncoding RNA Identification and Differential Expression Analysis

To obtain high−quality clean reads, raw reads were further filtered by fastp (v0.18.0). Clean reads were compared to the cow reference genomes with HiSAT2 (v2.1.0), and the transcripts were reconstructed with the software StringTie (v1.3.4, Computer Biology Center of Johns Hopkins University, Baltimore, MD, USA) to obtain known transcripts and new transcripts. The CNCI (v2) and CPC (v0.9−r2) (http://cpc.cbi.pku.edu.cn/ (accessed on 15 September 2021)) were used to assess the protein−coding potential of novel transcripts by default parameters. The intersection of both nonprotein−coding potential results was chosen as lncRNAs. lncRNA differential expression analysis was performed by DESeq2 software between the two different groups. To identify differentially expressed genes (DEGs) across samples or groups, the edgeR package (http://www.r−project.org/ (accessed on 15 September 2021)) was used. We identified differentially expressed transcripts with |log2fold change(FC)| > 1 and false discovery rate (FDR) < 0.05. 

### 2.6. Quantitative Real-Time Polymerase Chain Reaction (qRT-PCR) Validation

Total RNA was extracted with Trizol reagent (Invitrogen, Carlsbad, CA, USA). The density of samples’ RNA was quantified spectrophotometrically at 260/280 nm. The RNA was reverse transcribed using Prime ScriptTM RT Master Mix (TaKaRa, cat: RR036A, Otsu, Japan) according to the manufacturer’s protocols. The primers were designed with the NCBI Primer BLAST tool (https://www.ncbi.nlm.nih.gov/tools/primerblast (accessed on 1 August 2022)). The sequences of the primers of mRNAs and lncRNAs are listed in Table 1. All quantitative reverse transcriptase PCR (qRT−PCR) runs were performed on a StepOne Plus Real−Time PCR System (Applied Biosystems, Foster City, CA, USA) using ChamQ Universal SYBR qPCR Master Mix (Vazyme Biotech Co., Ltd., cat: Q711−02, Nanjing, China) in a reaction volume of 20 µL. The relative quantifications of mRNAs and lncRNAs were normalized to β−actin with the 2^−ΔΔCt^ method.

### 2.7. Function Enrichment Analysis

We selected the differential expression analyses of the lncRNAs. Gene ontology (GO) enrichment analysis and the Kyoto Encyclopedia of Genes and Genomes (KEGG) pathway analysis were performed to investigate the biological function of the target genes of DE lncRNAs using DAVID (http://david.abcc.ncifcrf.gov/ (accessed on 10 October 2021)). 

### 2.8. lncRNA-miRNA-mRNA Network Association Analysis

Based on our previous transcriptome sequencing results and small RNA sequencing results, we established ceRNA regulatory networks for DE lncRNAs. The ceRNA network was constructed based on ceRNA theory as follows: (1) the expression correlation between mRNA–miRNA or lncRNA–miRNA was evaluated using the Spearman rank correlation coefficient (SCC). Pairs with SCC <−0.7 were selected as negatively coexpressed lncRNA–miRNA pairs or mRNA–miRNA pairs, and both mRNA and lncRNA were miRNA target genes, and all RNAs were differentially expressed. (2) The expression correlation between lncRNA–mRNA was evaluated using the Pearson correlation coefficient (PCC). Pairs with PCC >0.9 were selected as coexpressed lncRNA–mRNA pairs, and both mRNA and lncRNA in these pairs were targeted and negatively coexpressed with a common miRNA. (3) A hypergeometric cumulative distribution function test was used to test whether the common miRNA sponges between the two genes were significant. As a result, only the gene pairs with a *p*−value less than 0.05 were selected.

## 3. Results

### 3.1. Comparison of Environmental Temperature and Humidity Index (THI) and Cow Information

**Figure 1:**
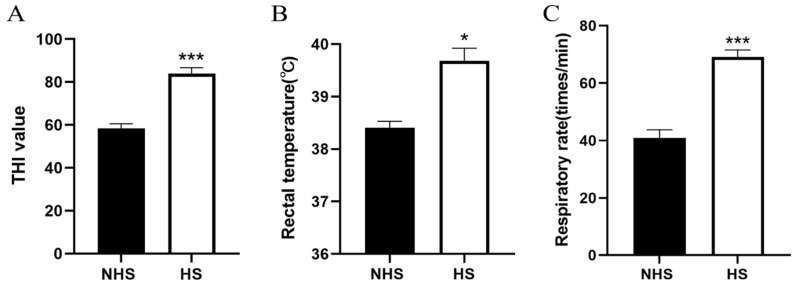
Comparison of environmental temperature and humidity index (THI) and cows’ information. (**A**) THI of cowshed. (**B**) Rectal temperature and (**C**) respiratory rate of cows between NHS and HS groups. * *p* < 0.05, *** *p* < 0.001. NHS, nonheat−stress group; HS, heat−stress group.

Researchers have shown that THI < 72 has no effect on dairy cows. It could cause slight heat stress in dairy cows between 72 and 78, moderate heat stress in dairy cows between 78 and 89, and severe heat stress in dairy cows above 90 [16]. In our study, the THI value of the HS group was significantly higher than that of the NHS group (Figure 1A, *p* < 0.001). Meanwhile, RT and RR of cows in the HS group were significantly higher than those in the NHS group (Figure 1B,C, *p* < 0.001). These results showed that the cows in the HS group were in a state of heat stress relative to the NHS group.

### 3.2. Effect of Heat Stress on Heat Shock Proteins and Antioxidant Enzymes in HPM-Axis-Related Tissues of Dairy Cows

**Figure 2:**
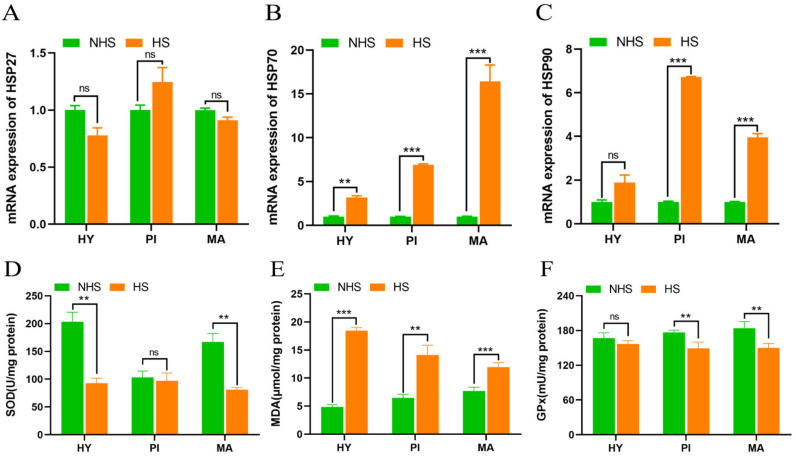
Effect of heat stress on heat shock proteins and antioxidant enzymes in HPM−axis−related tissues of dairy cows. (**A–C**) Relative mRNA expression of heat shock proteins (*HSP27*, *HSP70*, and *HSP90*), and (**D–F**) antioxidant enzyme activity in HPM−axis−related tissues between NHS and HS groups. Data are presented as the mean ± SEM (*n* = 3); ** *p* < 0.01, *** *p* < 0.001, ns *p* > 0.05. NHS, nonheat−stress group; HS, heat−stress group; SOD, superoxide dismutase; MDA, malondialdehyde; GPx, glutathione peroxidase; HY, hypothalamus; PI, pituitary; MA, mammary gland.

To evaluate the effect of heat stress on HPM−axis−related tissues of dairy cows, we detected the mRNA expression of heat shock protein. We found that the expression of HSP27 in HPM−axis−related tissues had no significant effect between NHS and HS groups (Figure 2A, *p* > 0.05). The expression of HSP70 was up−regulated (Figure 2B, *p* < 0.01) in HPM axis tissues of heat−stressed dairy cows. Moreover, the expression of HSP90 was significantly increased in pituitary and mammary glands (*p* < 0.01), but it had no significant effect in the hypothalamus (*p* > 0.05) under heat stress conditions (Figure 2C). Subsequently, we directly detected the content of antioxidant enzymes in HPM axis tissues. The results show that MDA content was significantly increased in HPM axis tissues between NHS and HS groups (Figure 2D, *p* < 0.01). SOD activity significantly decreased (Figure 2E, *p* < 0.01) in the hypothalamus and mammary gland, and GPx activity significantly decreased (Figure 2F, *p* < 0.01) in pituitary and mammary glands under heat−stress conditions.

### 3.3. Identification of lncRNAs in HPM-Axis-Related Tissues of Dairy Cows under Heat Stress

**Figure 3:**
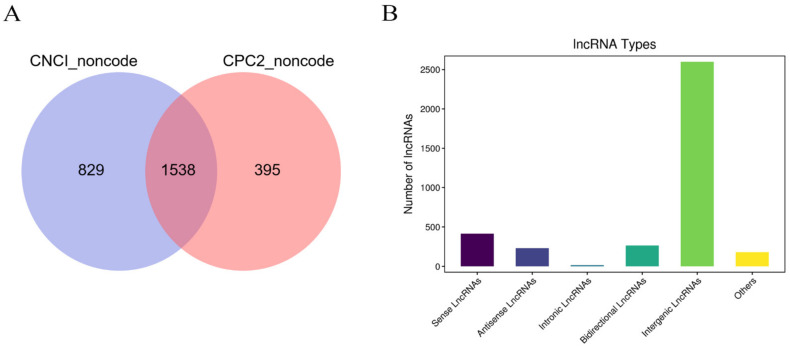
Identification of lncRNAs in HPM−axis−related tissues of dairy cows under heat stress. (**A**) Prediction of novel lncRNAs on the basis of the annotation results from the CNCI and CPC programs. (**B**) Classification of novel lncRNA types.

In the current study, 18 cDNA libraries in the HPM−axis−related tissues were constructed to identify the lncRNAs expressed in the NHS and HS groups. After the raw reads were subjected to quality control and filtering, high−quality filtered effective reads were obtained from the 18 libraries. The filtered reads were subsequently compared with the cow reference genome, and 81.08–85.63% reads were successfully mapped to the reference genome. Therefore, the DEG analysis based on the genome was considered reliable. A total of 2163 lncRNAs were detected in the NHS and HS, and 1538 novel lncRNAs were screened on the basis of the data from the CNCI and CPC2 programs (Figure 3A). We statistically analyzed the numbers of lncRNAs on the basis of their specific positions and found that intergenic lncRNAs accounted for 70.22% of all the lncRNAs (Figure 3B).

### 3.4. Differential Expression Analysis of lncRNAs

**Figure 4:**
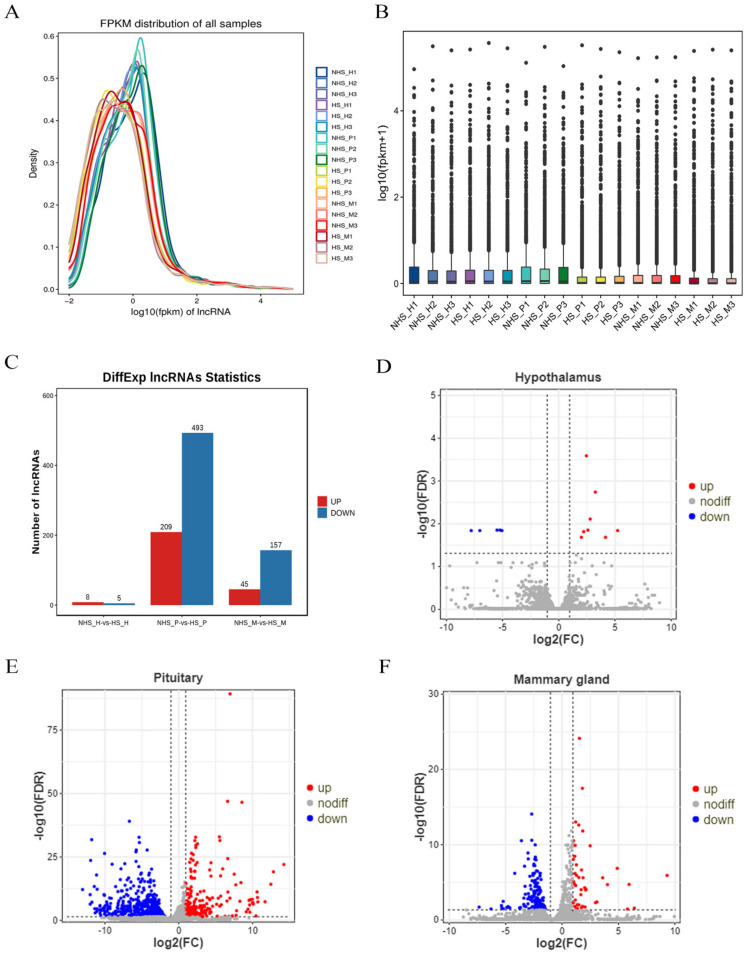
Differential expression analysis of lncRNAs. (**A**) Abundances of all transcripts in the 18 libraries. (**B**) Comparison of the overall expression levels between the NHS and the HS groups. (**C**) Differential expression of lncRNAs between the NHS and HS groups. (**D**) Volcano plot of DE lncRNAs in hypothalamus. (**E**) Volcano plot of DE lncRNAs in pituitary. (**F**) Volcano plot of DE lncRNAs in mammary gland. *p*−values and log2FC values were used to screen for differentially expressed transcripts according to the following thresholds: *p* < 0.05 and |log2FC| > 1. Red dots represent up−regulated lncRNAs and blue dots represent down−regulated lncRNAs. NHS, nonheat−stress group; HS, heat−stress group; H, hypothalamus; P, pituitary; M, mammary gland; FPKM, fragments per kilobase of exon model per million mapped fragments; FC, fold change; FDR, false discovery rate.

As shown in Figure 4A, we compared the expression abundance of all transcripts in 18 libraries. In general, the relative expression levels were lower in the NHS group than in the HS group, and the trend of down−regulation was obvious. The overall expression levels of HPM−axis−related tissues were also compared in the NHS and the HS groups (Figure 4B), and the expression levels in the HS group were slightly lower than those in the NHS group. Further, a total of 13, 702 and 202 DE lncRNAs were identified in the hypothalamus, pituitary, and mammary gland, respectively. Of lncRNAs, 8, 209 and 45 were up−regulated, and 5, 493 and 157 lncRNAs were down−regulated (Figure 4C–F).

### 3.5. Validation of DE lncRNAs with qRT−PCR

**Figure 5:**
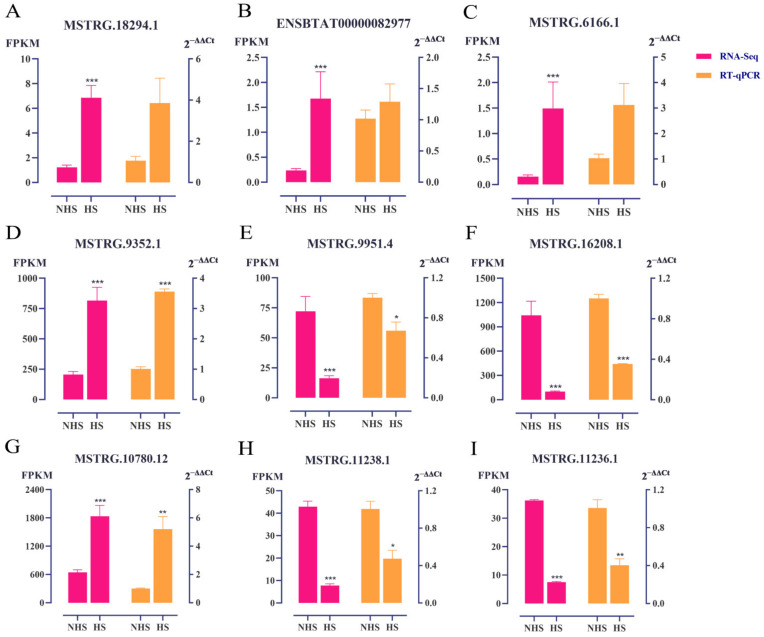
Comparison of the lncRNA expression levels determined by RNA−seq and qRT−PCR. The expression of lncRNAs in hypothalamus (**A−C**), pituitary (**D−F**), and mammary gland (**G−I**) in dairy cows under heat stress. The relative expression values were normalized to *β−actin* gene expression levels. Data are presented as the mean ± SEM (*n* = 3); * *p* < 0.05, ** *p* < 0.01, *** *p* < 0.001. NHS, nonheat−stress group; HS, heat−stress group; FPKM, fragments per kilobase of exon model per million mapped fragments.

We randomly selected three lncRNAs from HPM−axis−related tissues and detected their expression by qRT−PCR. The trends in the lncRNA expression changes were similar to those identified via RNA−seq, although some quantitative differences were found between the qRT−PCR and RNA−seq results (Figure 5). These findings indicated that the RNA−seq results were reliable and could be used for bioinformatic analysis.

### 3.6. Function Enrichment Analysis for Differentially Expressed lncRNAs

**Figure 6:**
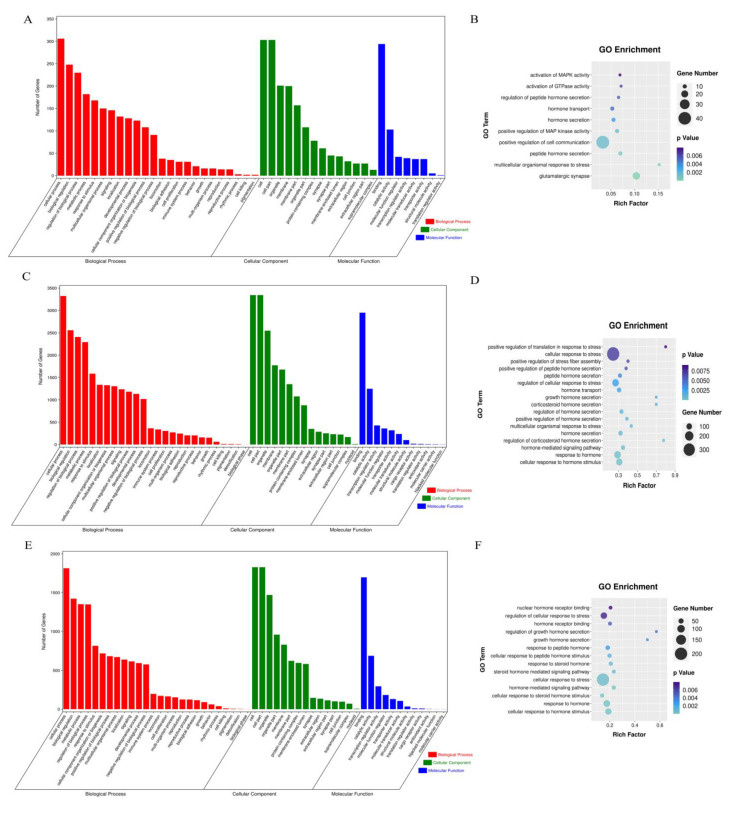
Gene ontology (GO) functional enrichment analysis on the target genes of DE lncRNAs in the hypothalamus (**A**,**B**), pituitary (**C**,**D**), mammary gland (**E**,**F**) in dairy cows under heat stress.

In order to determine the biological processes associated with the target genes of DE lncRNAs in HPM axis tissues, we performed GO and KEGG pathway enrichment analysis. In the hypothalamus, DE lncRNA target genes were significantly enriched in 504 GO pathways (*p* < 0.01), among which 357 GO pathways were associated with biological processes, 88 with cellular components, and 59 with molecular functions (Figure 6A, Table S1). The functional annotation by GO indicated that the predicted target genes in the hypothalamus were related to regulation of neurotransmitter levels, regulation of peptide hormone secretion, hormone transport, as well as multicellular organismal response to stress (Figure 6B).

In the pituitary, a total of 719, 173, and 162 GO terms (*p* < 0.01) were significantly enriched in biological processes, cellular components, and molecular functions, respectively (Figure 6C, Table S2). The functional annotation by GO indicated that the DE lncRNA target genes were related to cellular response to hormone stimulus, regulation of corticosteroid hormone secretion, multicellular organismal response to stress, as well as positive regulation of translation in response to stress (Figure 6D).

Moreover, DE lncRNA target genes were significantly enriched in 1432 GO terms (*p* < 0.01), among which 263 GO pathways were associated with biological processes, 108 with cellular components, and 115 with molecular functions in the mammary gland (Figure 6E, Table S3). The functional annotation by GO indicated that the DE lncRNA target genes were related to hormone−mediated signaling pathway, hormone receptor binding, and regulation of cellular response to stress (Figure 6F).

**Figure 7:**
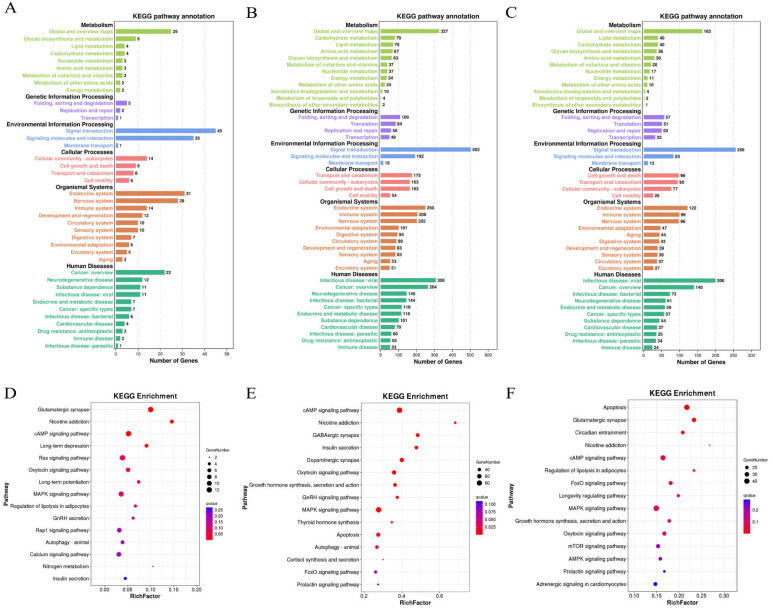
Kyoto Encylopedia of Genes and Genomes (KEGG) pathway analysis on the target genes of DE lncRNAs in the hypothalamus (**A**,**D**), pituitary (**B**,**E**), and mammary gland (**C**,**F**) in dairy cows under heat stress.

Furthermore, target genes of DE lncRNAs participate in energy metabolism, lipid metabolism, amino acid metabolism, signal transduction, cell growth and death, and environmental adaptation in the HPM−related tissues according to KEGG pathway enrichment analysis (Figure 7A–C). A total of 35, 104, and 52 KEGG pathways were significantly enriched in the HPM−related tissues, respectively (*p* < 0.05, Tables S4–S6). We found that the DE lncRNA target genes in the hypothalamus were enriched in glutamatergic synapse, cyclic adenosine monophosphate (cAMP) signaling pathway, mitogen−activated protein kinase (MAPK) signaling pathway, and autophagy (Figure 7D). In the pituitary, the DE lncRNA target genes were enriched in hormone−related pathways, such as growth hormone synthesis, secretion and action, thyroid hormone synthesis, cortisol synthesis and secretion, and prolactin signaling pathway (Figure 7E). The KEGG enrichment analysis of DE lncRNAs in the mammary gland showed that the meaningful pathways were related to apoptosis, such as the MAPK signaling pathway. Simultaneously, DE lncRNAs are also enriched in lactation−related pathways, such as mechanistic target of rapamycin (mTOR) signaling pathway, AMP−activated protein kinase (AMPK) signaling pathway, and prolactin signaling pathway (Figure 7F). From these analyses, we speculated that lncRNAs may participate in the stress response and physiological lactation process of dairy cows by regulating the expression of target genes.

### 3.7. lncRNA–miRNA–mRNA Regulatory Network Analysis

**Figure 8:**
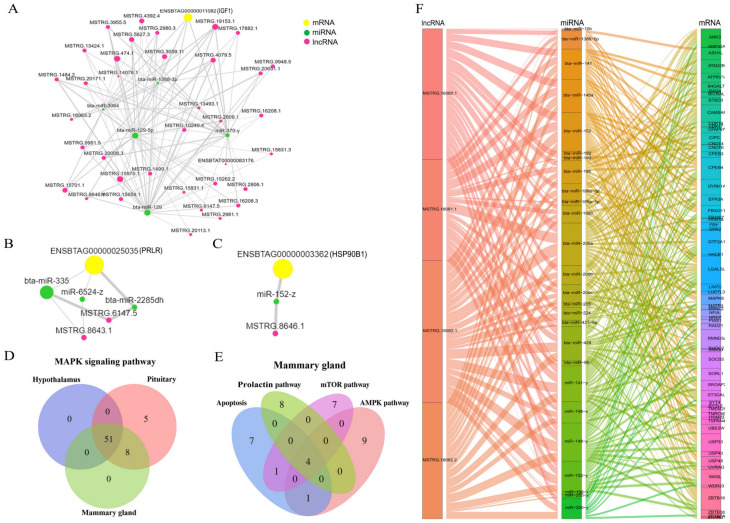
(**A**) The ceRNA regulatory subnetworks of IGF1. (**B**) The ceRNA regulatory subnetworks of PRLR. (**C**) The ceRNA regulatory subnetworks of HSP90B1. (**D**) Common lncRNAs involved in MAPK signaling pathway in HPM−axis−related tissues under heat stress. (**E**) Common lncRNAs involved in apoptosis, AMPK, mTOR, and prolactin signaling pathway in the mammary gland under heat stress. (**F**) The ceRNA regulatory networks of lncRNAs involved in apoptosis, AMPK, mTOR, and prolactin signaling pathway in the mammary gland under heat stress.

The ceRNA network was constructed to clarify the relationship among lncRNA, miRNA, and mRNA in HPM axis of dairy cows under HS. We first analyzed the ceRNA regulatory network of genes related to lactation and heat stress in the mammary gland. The ceRNA network of insulin−like growth factor 1 (IGF1, ENSBTAG00000011082) is shown in Figure 8A. IGF1 expression is regulated by 36 lncRNAs and 5 miRNAs and contains 106 lncRNA–mRNA pair ceRNA subnetworks (Table S7). The expression of prolactin receptor (PRLR, ENSBTAG00000025035) is regulated by two lncRNAs (MSTRG.6147.5, MSTRG.8643.1) and three miRNAs (bta−miR−2285dh, bta−miR−335, and miR−6524−z). Among them, MSTRG.6147.5/MSTRG.8643.1−bta−miR−335−PRLR were the main regulatory subnetworks (Figure 8B, Table S7). Moreover, heat shock protein 90 beta family member 1 (HSP90B1, ENSBTAG00000003362) was mainly regulated by MSTRG.8646.1 and miR−152−z (Figure 8C, Table S7).

Furthermore, in the previous KEGG enrichment analysis, we found that the MAPK signaling pathway was significantly enriched in three tissues. In order to further study the role of lncRNAs involved in the MAPK signaling pathway, the ceRNA network was constructed to clarify the relationship among lncRNA, miRNA, and mRNA in the MAPK signaling pathway of the HPM axis. In the three tissues, 51 lncRNAs participate in the MAPK signaling pathway (Figure 8D). ceRNA regulatory networks associated with these lncRNAs contain 3286 lncRNA–mRNA pairs. For instance, MAPK8 was regulated by 15 lncRNAs. The subnetworks of MSTRG.12225.1 and five miRNAs (bta−miR−10b, bta−miR−182, bta−miR−186, bta−miR−199a−3p, miR−200−x) may regulate the expression of MAPK8 (Table S8). 

Additionally, the KEGG enrichment analysis in the mammary gland found that apoptosis and lactation−related pathways, such as the AMPK, mTOR, and prolactin signaling pathway, were significantly enriched. Therefore, we analyzed the ceRNA network of lncRNAs involved in these pathways. We found that four common lncRNAs were involved in these pathways (Figure 8E). Meanwhile, ceRNA regulatory networks associated with these lncRNAs contain 772 lncRNA–mRNA pairs (Figure 8F, Table S9). Among them, suppressors of cytokine signaling (SOCS) 5 was regulated by MSTRG.16080.1, MSTRG.16082.1, MSTRG.16082.2, and MSTRG.16081.1, and three major miRNAs (bta−miR−1388−5p, bta−miR−141, and bta−miR−200a). Therefore, lncRNA may regulate the heat−stress response of dairy cows by regulating the key signal pathway of the HPM axis.

## 4. Discussion

In a previous study, we measured the serum endocrine hormones and biochemical indexes of cows in an NHS group and HS group. Heat stress significantly increased the levels of adrenocorticotropic hormone (ACTH) and cortisol (COR) in serum of dairy cows, but decreased the levels of triiodothyronine (T3) and thyroid hormone (T4). These results showed that the stimulation of the thermal environment caused the changes in the hypothalamic-pituitary-endocrine axis in dairy cows [14]. Further, in the current study, we used the transcriptome to analyze the differential expression profile of lncRNA in the HPM axis of heat−stressed dairy cows, and established the regulatory network of lncRNA–miRNA–mRNA in the hypothalamus, pituitary, and mammary gland. Due to limited funding, we could only randomly select the hypothalamus, pituitary, and mammary gland tissues of three cows from each group for transcriptome sequencing.

Heat shock proteins (HSPs) are polypeptides with a highly conserved structure, and are also called heat stress proteins since almost all organisms express heat shock protein when they are subject to extra stresses to a certain extent [17]. In order to determine the occurrence of heat stress in dairy cows, we detected the mRNA expression of *HSPs* in HPM−axis−related tissues. Our experiment found that *HSP70* was significantly up−regulated in hypothalamus, pituitary, and mammary gland tissues of heat−stressed dairy cows. Meanwhile, the expression of *HSP90* was significantly increased in pituitary and mammary glands. However, thermal stimulation did not cause a change in *HSP27* in HPM−axis−related tissues. HSP70 and HSP90, as ATP−dependent molecular chaperones, play a role in helping the folding of newly synthesized peptides, the assembly of multiprotein complexes, and the transport of proteins across cell membranes [18]. However, HSP27 has multiple phosphorylation sites and plays its role through protein phosphorylation [19]. Therefore, these results may be related to the molecular structure of HSPs. In addition, previous studies have shown that heat stress can cause antioxidant dysfunction in dairy cows, which is mainly manifested in the reduction of antioxidant enzyme activities, such as SOD and glutathione peroxidase (GSH−Px), and the increase in lipid peroxide MDA [20]. Some researchers mitigated the heat stress response of dairy cows by increasing the activity of antioxidant enzymes to improve the lactation performance [21,22]. In the present experiment, we found that MDA content was significantly increased in HPM axis tissues between NHS and HS groups. Meanwhile, SOD activity significantly decreased in the hypothalamus and mammary gland, and GPx activity significantly decreased in pituitary and mammary glands under heat stress conditions. These results demonstrated that the antioxidant capacity of HPM−axis−related tissues may be reduced, which indirectly proved that cows were in a heat−stress state. Therefore, the tissue samples we collected can be used for subsequent sequencing studies.

The mechanisms of noncoding RNAs (ncRNAs) in lactation and stress response have been studied, and they play an important role in regulating RNA transcription, protein translation, and protein interaction. Studies have confirmed that lncRNA plays an important role in lactation and heat stress response. In this study, 81.08–85.63% reads were successfully mapped to the reference genome through high−throughput sequencing. Further, a total of 13, 702, and 202 DE lncRNAs were identified from the hypothalamus, pituitary, and mammary gland, respectively. We selected 12 DE lncRNAs from HPM−axis−related tissues for validation. qRT−PCR results were highly consistent with those of RNA−seq. These results indicate that lncRNA may play a key role in the HPM axis of dairy cows after thermal environment stimulation. 

Differential lncRNA expression is often accompanied by changes in cellular function, and lncRNAs can interact with coding genes in both cis and trans manners to fulfill their functions [7]. We performed GO analysis and identified the biological processes related to stress response processes of DE lncRNA target genes in three tissues. The main functions include cellular response to stress, regulation of cellular response to stress, stress−activated MAPK cascade, regulation of response to stress, and stress−activated protein kinase signaling cascade. Subsequently, KEGG pathway enrichment analysis showed that target genes of DE lncRNAs were closely related to stress response and physiological lactation processes. For instance, the target genes of DE lncRNAs in the hypothalamus were highly enriched in the oxytocin signaling pathway, gonadotropin−releasing hormone (GnRH) secretion, and insulin secretion. In the pituitary, the target genes of DE lncRNAs were also mainly enriched in hormone−related pathways, such as cortisol synthesis and secretion, thyroid hormone synthesis, growth hormone synthesis, secretion and action, and prolactin signaling pathway. COR, as the most sensitive hormone in the stress response, increases significantly after heat stimulation [23,24]. Researchers also found that the effects of short−term and long−term environmental heat on COR were obvious, initially increased due to acute stressors, and plasma levels decreased after long−term exposure to stressors [25]. Moreover, thyroid hormone plays a key role in regulating body temperature, energy intake, and metabolism [26]. The stimulation of the thermal environment can significantly reduce the secretion of thyroid hormone to regulate the thermal homeostasis of dairy cows [27]. Universally, prolactin (PRL) and growth hormone (GH) are essential in maintaining the physiological process of lactation. The study has shown that PRL and GH in hormone−response pathways are involved in energy metabolism during thermoregulation processes in cattle, and single nucleotide polymorphisms (SNPs) within genes of the PRL and GH pathways are predictors of reproductive phenotype in heat−stressed Holstein cows [28]. Thus, these results indicate that lncRNA may be involved in regulating the expression of hormone−related target genes in response to thermal stimulation. Simultaneously, the meaningful pathways of DE lncRNA target genes in the mammary gland were related to apoptosis, mTOR signaling pathway, and AMPK signaling pathway. Chen et al. found that heat stress inhibits the proliferation of BMECs, aggravates cellular oxidative stress, causes mitochondrial dysfunction, and promotes BMEC apoptosis [29]. Meanwhile, sirtuin 3 (Sirt3) can protect BMECs from heat stress damage through the AMPK signaling pathway [30]. These studies support our findings that lncRNAs may play an important role in heat−stress−induced cell damage. In addition, the AMPK−mTOR pathway is closely related to cell energy and material metabolism [31]. Some studies have shown that the AMPK−mTOR pathway is involved in regulating the synthesis of casein in mammary cells [31,32]. Liang et al. analyzed and predicted the lncRNAs related to milk fat metabolism, and found that the target genes of these lncRNAs were also significantly enriched in MAPK, AMPK, and mTOR signaling pathways [33]. Interestingly, we found that DE lncRNAs were significantly enriched in the MAPK signaling pathway in the hypothalamus, pituitary, and mammary gland in the current study. The MAPK family is a group of serine/threonine kinases, which can be activated by cytokines, neurotransmitters, hormones, and cell stress [34]. Accumulating evidence has indicated that thermal stimulation induces MAPK activation, thereby affecting cell proliferation and apoptosis [35,36]. Consequently, we speculate that the differential expression of lncRNAs in the HPM axis may play an important role in regulating the lactation process of heat−stressed dairy cows. These possible associations should be further explored in future study.

MicroRNA (miRNA), as a highly conserved small molecule ncRNA, can regulate various physiological and pathological processes [37]. Many studies have shown that lncRNAs have miRNA−response elements (MREs), which can act as competitive endogenous RNA (ceRNA) to regulate miRNAs and mRNAs, thereby exerting their biological functions [38,39]. For example, lncRNA AK017368 promotes proliferation and inhibits differentiation of myoblast cells by attenuating the function of miR−30c [40]. Liu et al. found that lncRNA−MEG3 is highly expressed in bovine skeletal muscle tissue, and lncRNA−MEG3 can promote bovine skeletal muscle differentiation by interacting with miRNA−135 and myocyte enhancer factor 2C (MEF2C) [41]. Additionally, CTTN−IT1 is a novel lncRNA that is a ceRNA of miR−29a and can promote skeletal muscle satellite cell proliferation and differentiation by restoring the expression of Yes−associated protein 1 (YAP1) when it is inhibited by miR−29a in Hu sheep [42]. In our present study, some ceRNA regulatory networks were established in the HPM axis of dairy cows under heat stress, such as MSTRG.8646.1−miR−152−z−HSP90B1, MSTRG.12225.1−bta−miR−182−MAPK8, and MSTRG.17399.2−bta−miR−186−MAPK8. In addition, the expression of activating transcription factor 2 (ATF2) was regulated by MSTRG.17501.1 and bta−miR−1388−5p. Additionally, MSTRG.4129.1 and two miRNAs (bta−miR−200b and miR−200−y) also regulate the expression of ATF2. In a previous study, stress response activated ATF2 transcription, which is related to apoptosis [43]. In mammary glands, we also found ceRNA networks involved in lactation−related genes and pathways, such as MSTRG.6147.5/MSTRG.8643.1−bta−miR−335−PRLR, MSTRG.16080.1−bta−miR−200a−SOCS5, MSTRG.16081.1−bta−miR−1388−5p−SOCS5, and MSTRG.16082.2−bta−miR−141−SOCS5. IGF1 expression was regulated by 36 lncRNAs and 5 miRNAs. PRLR and IGF1 are involved in regulating the growth and development of mammary glands and milk secretion [44,45]. The Janus kinase–signal transducer and activator of transcription (JAK−STAT) signaling pathway and suppressors of cytokine signaling (SOCS) family genes play a crucial role in controlling cytokine signals in the mammary gland and thus mammary gland development [46]. Accordingly, we speculated that cows may experience changes in the main lactating pathway to respond to the thermal environment by regulating the expression of lncRNA and ceRNA (lncRNA–miRNA–mRNA) networks in the HPM axis. The specific mechanism should be studied in future research.

In conclusion, the current study indicated that the expression profiles of lncRNAs in HPM−axis−related tissues of heat−stressed and nonheat−stressed cows were different. Moreover, cows may experience changes in the main lactating pathway to respond to the thermal environment by regulating the ceRNA (lncRNA–miRNA–mRNA) networks in the HPM axis. Consequently, these results provide a theoretical basis for the molecular mechanism of the stress response and lactation physiology of HPM−axis−related tissues under HS.

## Figures and Tables

**Table 1:** Primer sequences of mRNAs and lncRNAs.

RNAs	Forward (5′–3′)	Reverse (5′–3′)
HSP27	CCATTCCCGTCACCTTCCA	CGCTGGGCTAAGGGTCTTTAC
HSP70	CTGAACCCGCAGAACACG	CCTTGGTCTCCCCTTTGT
HSP90	TGACCAGCACCTACGG	CACCAGGTCCTTGACG
MSTRG.18294.1	CTAAACAGCAGCAACAGTGAGC	GGAAAGATTGAGGGCAGGA
ENSBTAT00000082977	AGGTCTTGGAGGCATCTTATC	CGCATCACCAGGACGAAT
MSTRG.6166.1	GAAGACACCACCATCGGAGA	CACCTGCCACAACGAAGAGTT
MSTRG.9352.1	CTTTAGCATCATTCCTTCCACC	GGGATTATGACTGAACGCCTC
MSTRG.9951.4	TGGGTCTTGCTGGATGCTC	TGTGACGGGTTCCCTGTTG
MSTRG.16208.1	GATTGTGAGGCATCGTCC	AGGGCTGTCAGATTCAAGG
MSTRG.10780.12	CCATTGATCGCCAGGGTT	TTCGGGAGGGACGCACAT
MSTRG.11238.1	GTTTGGGTCTTTCCCTACTGC	ATGTGCCAAGTAACAGAGAAGC
MSTRG.11236.1	TGGGTCTTTCCCTACTGCTCT	ATAAGTGGCTGATGTGCCAAG
β−Actin	TCACCAACTGGGACGACA	GCATACAGGGACAGCACA

## Data Availability

All raw transcriptome data reported in current article have been deposited at the National Center for Biotechnology Information (https://www.ncbi.nlm.nih.gov/sra/PRJNA885909 (accessed on 30 September 2022)).

## Supplementary Materials

The following supporting information can be downloaded at: https://www.mdpi.com/article/10.3390/biom13020390/s1, Table S1: GO terms of DE lncRNA target genes in the hypothalamus of dairy cows under HS; Table S2: GO terms of DE lncRNA target genes in the pituitary of dairy cows under HS; Table S3: GO terms of DE lncRNA target genes in the mammary gland of dairy cows under HS; Table S4. KEGG pathways of DE lncRNA target genes in the hypothalamus of dairy cows under HS; Table S5. KEGG pathways of DE lncRNA target genes in the pituitary of dairy cows under HS; Table S6. KEGG pathways of DE lncRNA target genes in the mammary gland of dairy cows under HS; Table S7. The ceRNA regulatory network in the mammary gland of dairy cows under HS; Table S8. The ceRNA networks of DE lncRNA in MAPK signaling pathway in HPM axis; Table S9. The ceRNA networks of DE lncRNA target genes enriched in apoptosis, AMPK, prolactin, and mTOR signaling pathway in mammary gland.
